# Orthodontic Treatment of a Patient With Non-Syndromic Oligodontia and a Skeletal Class Ⅲ Relationship: A Case Report and Six Years’ Follow-Up

**DOI:** 10.7759/cureus.62563

**Published:** 2024-06-17

**Authors:** Ryo Kunimatsu, Yuki Asakawa, Ayaka Nakatani, Shuzo Sakata, Kotaro Tanimoto

**Affiliations:** 1 Department of Orthodontics and Craniofacial Development Biology, Graduate School of Biomedical & Health Sciences, Hiroshima University, Hiroshima, JPN

**Keywords:** skeletal class ⅲ relationship, orthodontic treatment, partial edentulism, congenitally missing teeth, oligodontia

## Abstract

Partial edentulism, characterized by the congenital absence of six or more permanent teeth (oligodontia), excluding the third molars, manifests with variable maxillofacial skeletal morphologies and occlusions, depending on the site and number of missing teeth, complicating treatment planning for occlusion and gain of function. Herein, we describe the case of a patient with seven non-syndromic congenitally missing permanent teeth (four in the maxillary and three in the mandibular dentition, excluding the third molars), who underwent orthodontic treatment, restorative procedures, and long-term follow-up for six years. The patient was an 18-year-old man presenting with a chief complaint of congenital absence of some permanent teeth and dental malalignment on the first visit. The mandibular right central incisor, bilateral mandibular second premolars, bilateral maxillary lateral incisors, and bilateral maxillary canines were congenitally absent, while the deciduous maxillary lateral incisors, maxillary canines, and mandibular second molars were over-retained bilaterally. Since the persisting deciduous teeth were remarkably well preserved, the patient was willing to retain them as far as possible; thus, we chose orthodontic and restorative treatment to preserve the deciduous teeth. Occlusion was established after the initiation of dynamic orthodontic treatment; restorative treatment with resin-based materials was performed for the bilateral maxillary deciduous incisors, bilateral maxillary deciduous canines, and bilateral mandibular second primary molars after bracket removal, and the retention phase of orthodontic treatment was initiated. At present, six years after establishing retention, the patient exhibits a good occlusal relationship. It is difficult to achieve complete space closure using orthodontic treatment alone in cases with six or more congenitally missing permanent teeth. In addition to considerations for age, esthetic issues due to missing permanent teeth, and maxillofacial skeletal morphology, it is necessary to preserve the deciduous teeth as much as possible and ensure multidisciplinary medical cooperation, including the transition to prosthodontic treatment during long-term follow-up.

## Introduction

Congenital absence of permanent teeth is the most frequent congenital anomaly in the maxillofacial region [1,2]. Tooth loss leads to incorrect inclination of critical teeth, extrusion of the occlusal antagonist in the opposing dentition, displacement of the maxillomandibular midline, atrophy of the alveolar ridge, and malocclusions, such as hyperocclusion and interdental spaces, resulting in functional masticatory and phonetic disorders [3-5]. Since April 2012, “non-syndromic partial edentulism with congenital absence of six or more permanent teeth” has been considered a specific disease entity, which is covered by orthodontic treatment-related insurance benefits in Japan. In this milieu, the number of patients with non-syndromic partial edentulism visiting orthodontists' clinics in Japan has reportedly increased due to the provision of insurance coverage for treatment. However, depending on the site and number of congenitally missing permanent teeth, treatment planning for establishing occlusion and gain of function is complicated by variations in the maxillofacial skeletal morphology [6]. Therefore, there is an urgent need for knowledge and clarification of orthodontic planning and implementation for patients with non-syndromic partial edentulism. Non-syndromic oligodontia is a rare congenital disorder, and its treatment can be an arduous task. The principal goal of management is to improve esthetics and mastication. Moreover, treatment often requires a multidisciplinary approach. The purpose of this study was to report a case of long-term follow-up of orthodontic treatment, including restorative treatment, for a patient with non-syndromic congenital absence of seven permanent teeth, and review the relevant literature.

## Case presentation

**Figure 1:**
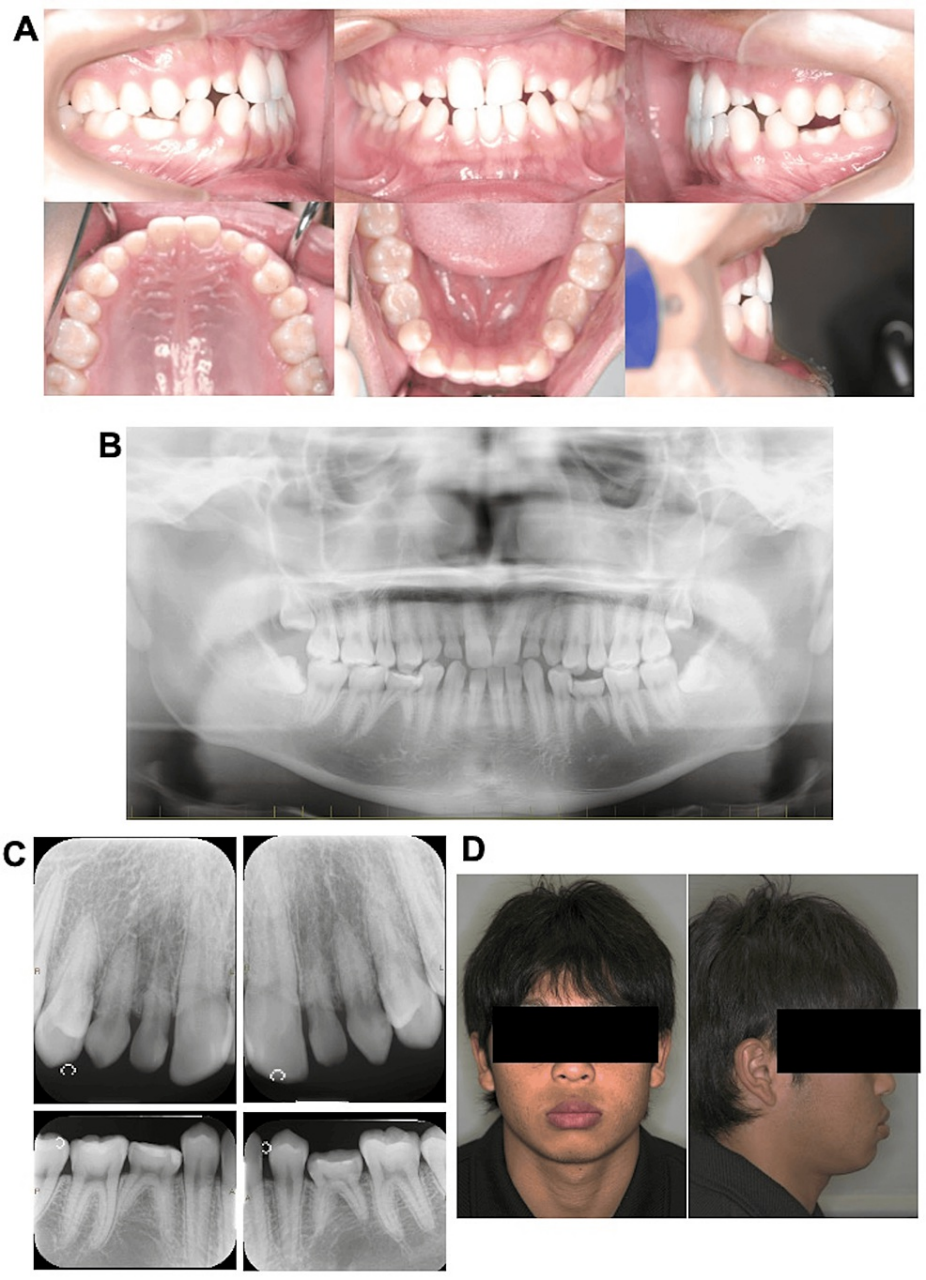
Initial examination A: pretreatment intraoral photographs; B: pretreatment panoramic radiograph; C: pretreatment intraoral radiograph; D: pretreatment facial radiograph

An 18-year-old man visited our hospital owing to the congenital absence of some permanent teeth and dental malalignment (i.e., malocclusion of the teeth). Around the age of nine years, he visited a general dentist, who noted that some permanent teeth were congenitally absent. Between nine and 10 years of age, improvement of the reverse tegmentum of maxillary left central coaptation of the sexual arch was performed at another hospital. This intervention was followed by subsequent monitoring. He was referred to our hospital with the understanding that the treatment for the congenital absence of six or more permanent teeth would be covered by insurance. The intraoral findings included a crown fracture of the maxillary left central incisor due to trauma. The mesiodistal positioning of the maxillary and mandibular first molars showed a bilateral angle class I relationship with an overjet of 0.6 mm and overbite of 0.2 mm (Figure 1A). On clinical examination, the mandibular right central incisor was missing, and interdental spaces were observed between the upper and lower anterior teeth due to missing teeth. The deciduous maxillary lateral incisors, maxillary canines, and mandibular second molars were over-retained bilaterally (Figure 1A). The right and left deciduous mandibular second molars were infra-occluded, and the right maxillary first premolar, right maxillary second premolar, and bilateral mandibular canines were supra-occluded. Panoramic and intraoral radiography revealed the presence of the following deciduous teeth: bilateral maxillary lateral incisors, bilateral maxillary canines, and bilateral mandibular second molars; the permanent mandibular left central incisor was present, and the bilateral mandibular second premolars were completely absent (Figure 1B, 1C). Root resorption of the persisting deciduous teeth was mild (Figure 1B, 1C). Third molars were observed bilaterally in the upper and lower jaws (Figure 1B, 1C). 

**Figure 2:**
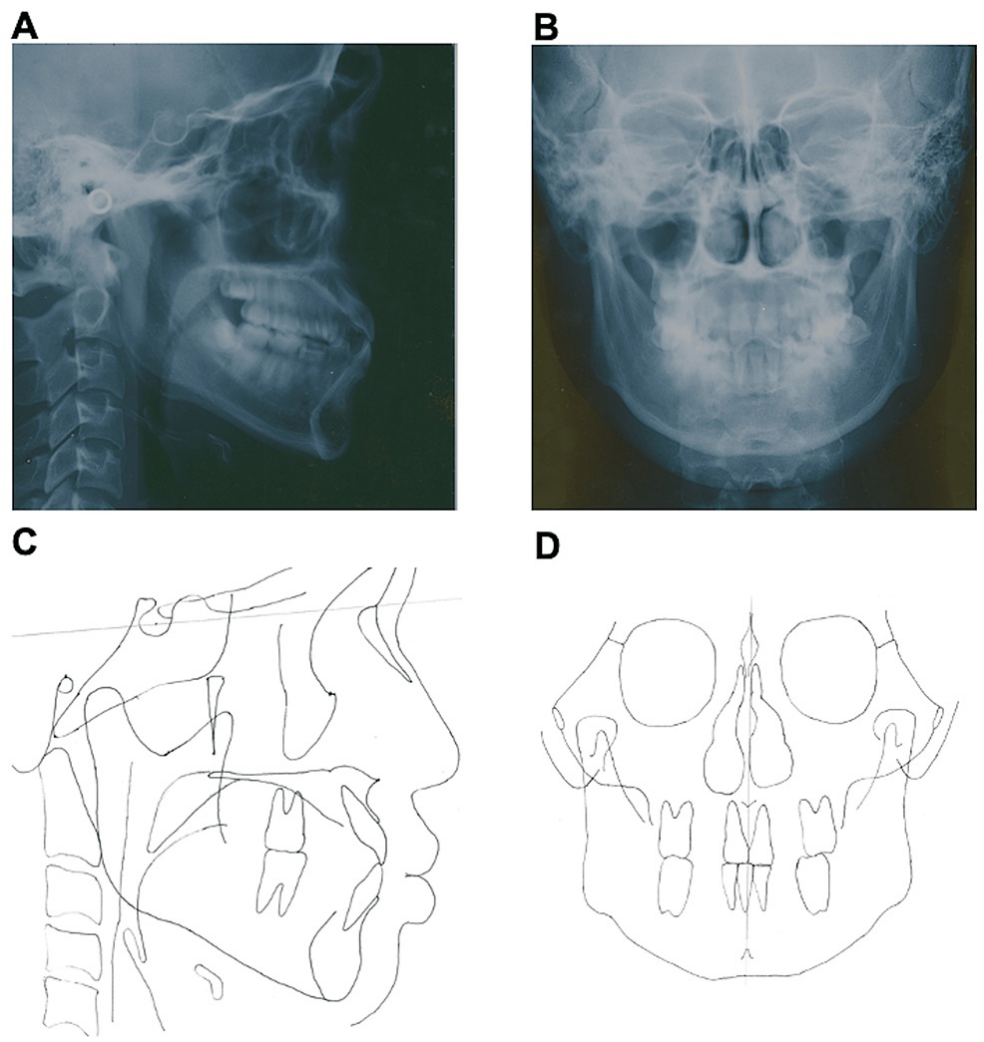
Pretreatment cephalometric radiography and cephalometric tracing A: pretreatment lateral cephalometric radiograph; B: pretreatment frontal cephalometric radiograph; C: pretreatment lateral cephalometric tracing; D: pretreatment frontal cephalometric tracing

Facial photography revealed bilateral symmetry with a short facial form, and the facial profile was concave (Figure 1D). Lateral cephalography revealed standard radiographic findings, with an SNA (sella, nasion, point A) angle of 82.6°; the relationship of the maxilla to the skull was within the standard range. Conversely, the considerably large SNB (sella, nasion, point B) angle measuring 82.6° and low ANB (point A, nasion, point B) angle measuring 0.1° indicated a skeletal class III relationship (Figure 2A, 2C; Table 1). Moreover, protrusion of the mental region was observed, as evidenced by the large facial angle. A low angle was observed vertically, due to the smaller Y-axis, mandibular plane angle (SN-MP), and Frankfort-mandibular plane angle (FMA). In the dentition, the inclination of the upper and lower central incisors was within normal limits, as the (upper incisor) U1 to sella-nasion (SN) angle was 111.6° and the Frankfort-mandibular incisor angle (FMIA) was 64.4° (Table 1). Frontal cephalometric radiography revealed no deviation of the maxilla and mandible with respect to the facial midline (Figure 2B, 2C). 

**Table 1:** Values obtained after lateral cephalometric analysis at the first visit SNA: angle formed from the points sella (S), nasion (N), and A; SNB: angle formed from the points S, N, and B; FMA: Frankfort-mandibular plane angle; SN-MP: Sella-Nasion to the mandibular plane angle; IMPA: angle formed by extending the lower incisor long axis to the mandibular plane; FMIA: Frankfort-mandibular incisor angle

Location	Pretreatment	Norm
SNA	82.7°	82.8 ± 2.2°
SNB	82.6°	79.5 ± 2.0°
ANB	0.1°	3.0 ± 1.3°
Facial angle	89.3°	85.1 ± 3.2°
Y-axis	60.2°	65.5 ± 3.0°
FMA	22.4°	25.9 ± 5.9°
SN-MP	29.8°	32.5 ± 4.9°
Gonial angle	122.3°	119.6 ± 6.9°
U1 to SN	111.6°	109.1 ± 6.8°
IMPA	93.2°	94.0 ± 9.3°
FMIA	64.4°	60.1 ± 10.2°
Interincisal angle	125.4°	123.9 ± 8.1°
U1 to A-Pog (mm)	6.8	9.6 ± 1.8
L1 to A-Pog (mm)	6.3	4.6 ± 2.5
E-line: upper lip (mm)	2.3	2.3 ± 1.3
E-line: lower lip (mm)	6.0	1.7 ± 1.8
Overjet	0.6	3.1 ± 1.1
Overbite	0.2	3.3 ± 1.9

Written informed consent was obtained from the patient. Approval by the ethics committee was not required as the study involved a single case report.

Diagnosis and treatment planning

The patient was diagnosed with skeletal mandibular prognathism with over-retained deciduous bilateral maxillary lateral incisors, bilateral maxillary canines, and bilateral mandibular second molars, in addition to missing bilateral mandibular second premolars, mandibular right central incisor, and interdental spaces in the upper and lower anterior segments due to missing teeth. For this case, three possible treatment plans were formulated. The first included orthodontics and restorations, while preserving the deciduous teeth as much as possible; the second entailed orthodontic treatment with the extraction of the deciduous teeth, prosthodontic replacement for the missing/extracted teeth, and mesial movement of the upper and lower molars. The second plan entailed extracting the over-retained deciduous teeth, i.e., the bilateral maxillary canines, and mandibular second molars. The maxillary central incisors showed a proclination of 2.4 mm and the lingual inclination of the left mandibular central incisor was 0 mm. Orthodontic anchorage screws were to be used in conjunction with multibracket appliances in the upper and lower jaws, along with sequential extraction of individual teeth to achieve mesial movement of the maxillary and mandibular molars (maxillary molar, 5.9 mm; mandibular molar, 6.7 mm). The space created by the extraction of the bilateral deciduous maxillary lateral incisors, amounting to 7.0 mm, would be managed by prostheses. Replacement of the mandibular right central incisor region would require a space of 6.0 mm, and the maxillary and mandibular midlines would be corrected using prosthetic procedures. 

The third treatment option entailed a plan of orthodontic treatment combined with jaw surgery. For preoperative orthodontic treatment in the maxillary dentition, the maxillary bilateral deciduous lateral incisors would be extracted, which would permit shifting the maxillary central incisors 1.0 mm palatally, and reduce the migration and width-diameter of the maxillary molars for alignment. In the mandibular dentition, a space of 5.4 mm for the replacement of the mandibular right central incisor and 1.0 mm labial inclination of the mandibular left central incisor would be needed. The bilateral mandibular third molars would be extracted. After orthodontic treatment, the mandible would be moved posteriorly by 4.4 mm using sagittal split osteotomy. After postoperative orthodontic treatment, the space in the mandibular right central incisor region would be closed with a prosthesis. The mandibular bilateral deciduous canines and mandibular bilateral second deciduous molars would be preserved in a classic fashion, and if they could not be conserved in the future, prosthetic treatment would be planned.

The plan chosen for orthodontic treatment included the restoration and preservation of the deciduous teeth as much as possible. The evaluation revealed that the maxillary central incisors showed a labial inclination of 2.1 mm, and the mandibular central incisor was tilted lingually by 0.5 mm. In order to maintain the angle class I maxillomandibular molar relationship, fixed multibracket appliances were used to improve the malalignment of individual teeth, and the deciduous bilateral maxillary incisors, maxillary canines, and mandibular second molars were restored using resin-based materials. In addition, management of the mandibular right-sided central incisor defects was attempted using the final row of sleeve insiders. On the basis of the comparison and examination of the above-mentioned treatment plans, our first choice was to perform orthodontic treatment while retaining the deciduous teeth because they were very well preserved, as well as achieving a class I occlusal relationship between the maxillary and mandibular molars. Moreover, the patient wanted dental treatment with orthodontics and restorations while preserving the deciduous teeth as much as possible, without surgery.

Course of treatment

**Figure 3:**
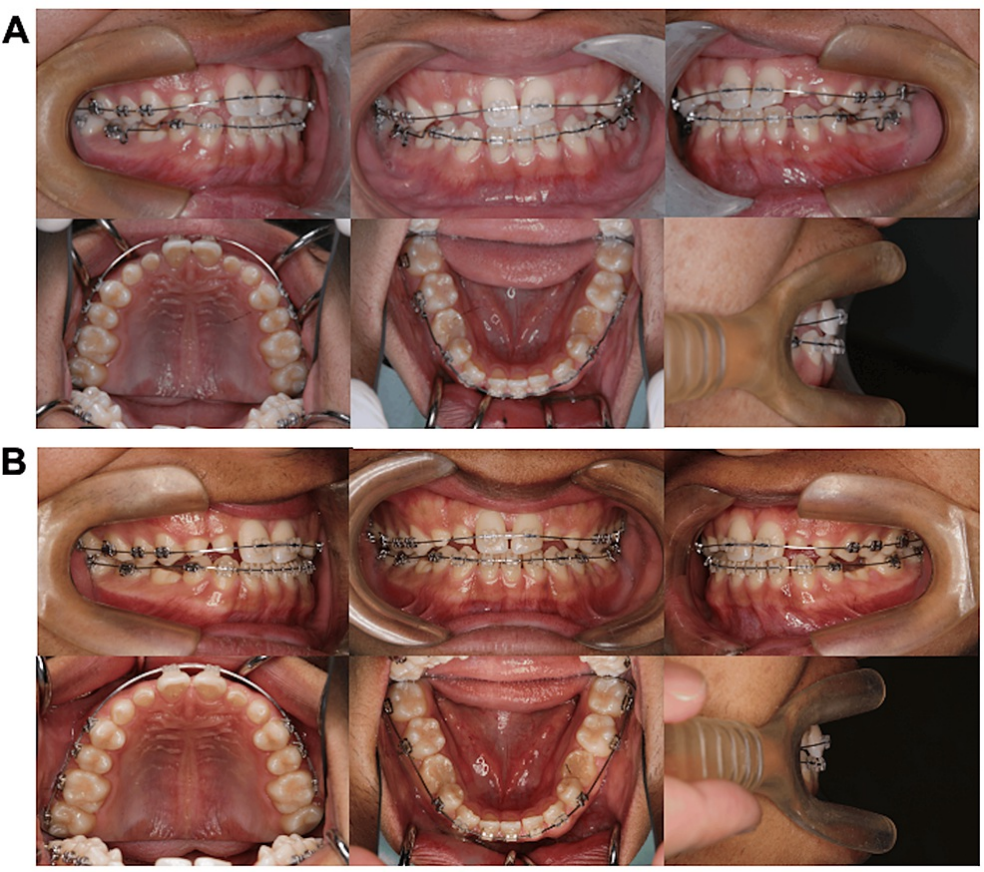
Intraoral photographs acquired during the orthodontic treatment A: intraoral photograph obtained four months after starting orthodontic treatment; B: oral photograph obtained eight months after starting orthodontic treatment.

Multi-bracket appliances (0.018 × 0.025-inch edgewise brackets) were attached to the upper and lower permanent teeth, and alignment was initiated with Ni-Ti archwires. The multibracket appliance was not placed on the residual deciduous teeth to preserve them as much as possible, and no orthodontic mechanical system was fixed over these teeth throughout the treatment period. Four months after the initiation of orthodontic treatment, the right and left maxillary premolars, which were extruded, interfered occlusally with the wires; thus, β-titanium archwires were used to connect the individual teeth (Figure 3A). A 0.014-inch β-titanium archwire was bent downward to prevent occlusion and to connect the individual teeth with the wire at the site of occlusion of the archwire (Figure 3A). At that time, in order to prevent labial proclination of the mandibular anterior teeth and occlusal relationship of the anterior teeth, a stop-loop bend was placed on the mesial part of the first molar and tie-back was performed, and the process of leveling was continued while maintaining the long diameter of the mandibular dental arch. Since the patient complained of pain due to orthodontic treatment at the leveling stage, we decided to adjust the force system to light forces as much as possible and refrain from using a large size rectangular (e.g., 0.017 × 0.025-inch and 0.018 × 0.025-inch) wire.

**Figure 4:**
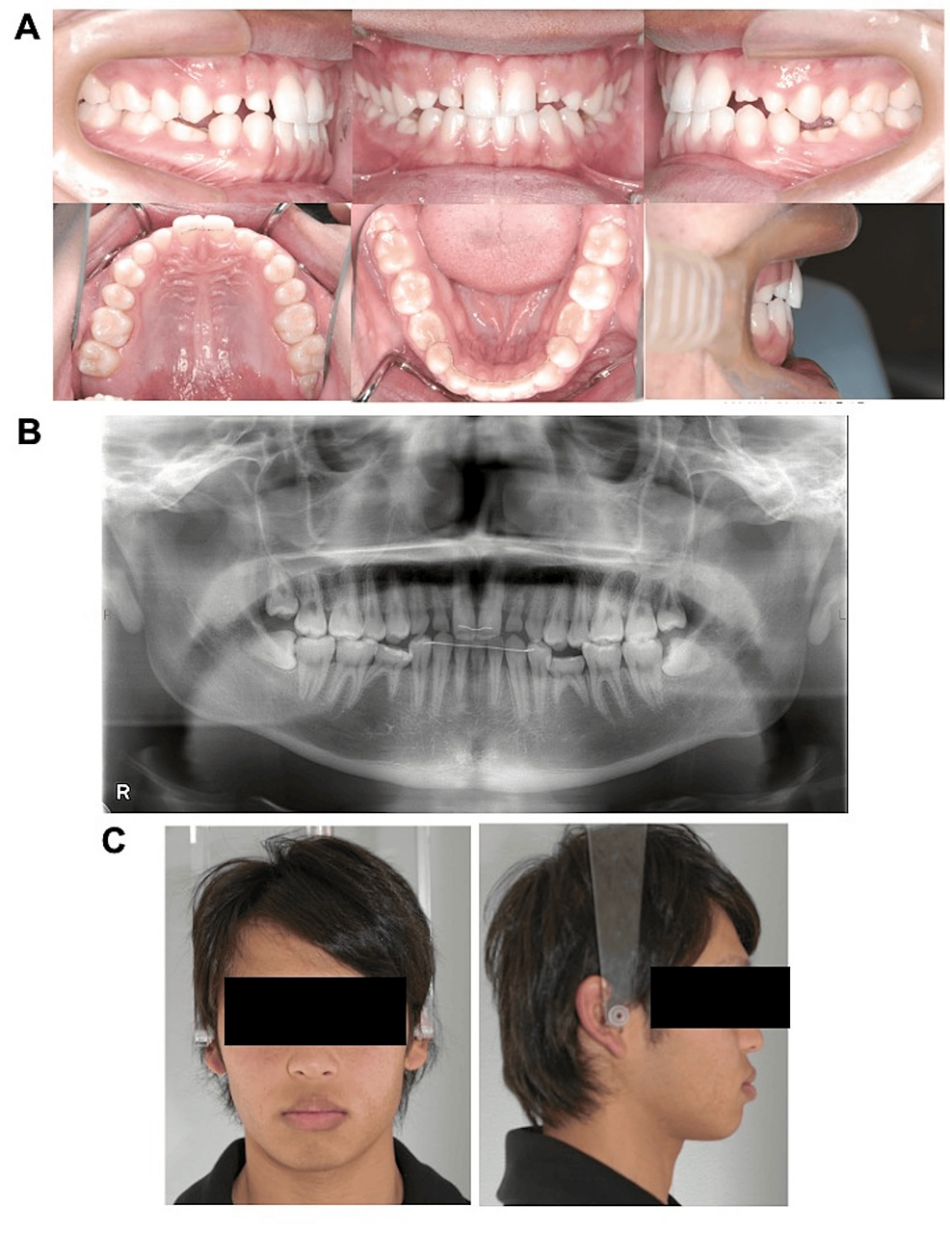
Posttreatment intraoral photograph, facial photograph, and panoramic radiographs A: intraoral photograph obtained at the end of orthodontic treatment; B: panoramic radiography performed at the end of the orthodontic treatment; C: facial photograph obtained at the end of orthodontic treatment.

**Figure 5:**
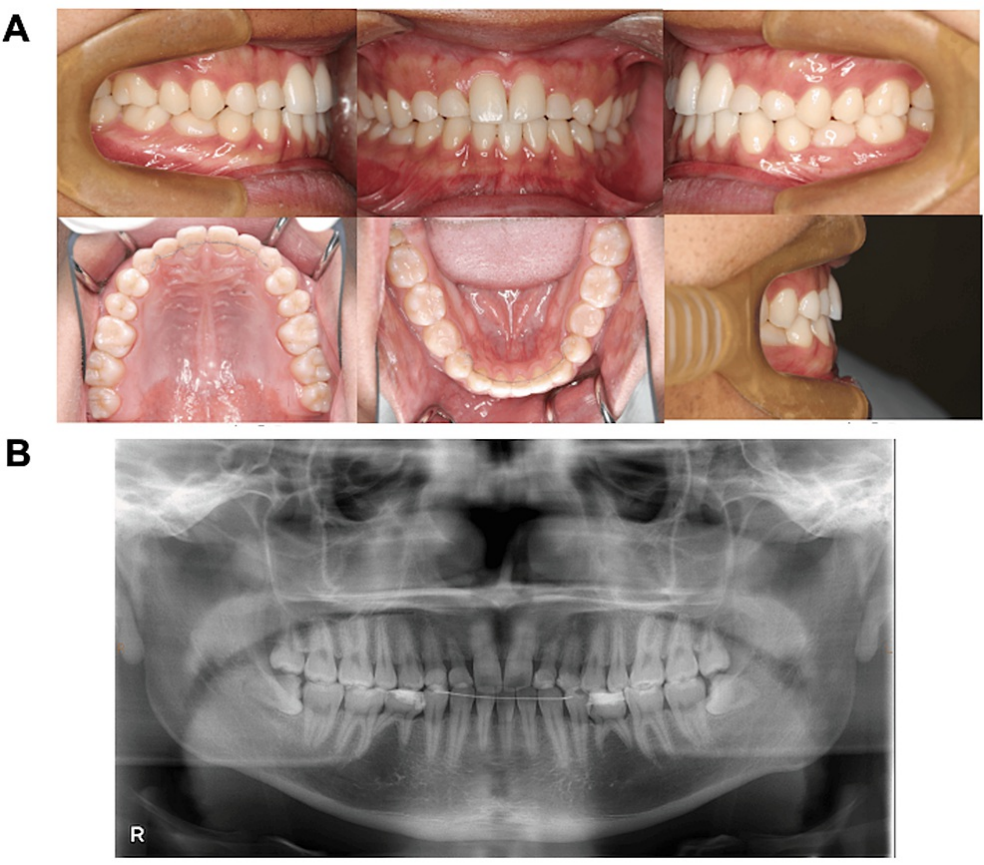
Intraoral and panoramic radiographs acquired after the conservative restorative treatment A: intraoral photographs; B: panoramic radiography

Eight months after the initiation of orthodontic treatment, tooth alignment was completed and the extrusion of the maxillary bilateral first premolars was also improved; therefore, a 0.016 × 0.022-inch cobalt-chromium archwire was mounted to obtain the ideal arch form, and an intimate occlusion was established (Figure 3B). The intended occlusal relationship was established 13 months after the initiation of orthodontic treatment, and the patient’s satisfaction with the dentition status was confirmed. Thereafter, a palatal-bonded dead retainer spanning the maxilla and a lingual-bonded spanning the central incisors of the right and left mandibular first premolars were fixed (Figure 4A). After debonding the brackets, conservative resin restorations were performed bilaterally for the deciduous maxillary lateral incisors, maxillary canines, and mandibular second molars (Figure 5A). After the completion of restorative treatment, the previous retainer was replaced with a palatal-bonded retainer connecting the maxillary deciduous canines, while a wrap-around retainer was attached to the maxilla and mandible, and the retention phase was continued. Compared with the facial photographs at the initial examination, there were no noticeable changes in the facial features (Figure 4C).

As a strategy for maintenance therapy, a lingual-bonded retainer was installed to prevent posterior movement of the maxillary and mandibular anterior teeth. In order to prevent relapse of the entire maxillary and mandibular dentition, the patient was advised to use a removable retainer every night for two years after the completion of multibracket treatment.

After the completion of multibracket treatment, the patient was asked to visit the hospital every three months for one year to check the bonded retainers’ fit status and receive instructions on the use of the retainers, as well as to check the status of the primary teeth, the remaining dentition, and oral hygiene. After the second year of retention, the patient was asked to visit the clinic every six months for follow-up.

Changes after treatment

Changes after Orthodontic Treatment

**Figure 6:**
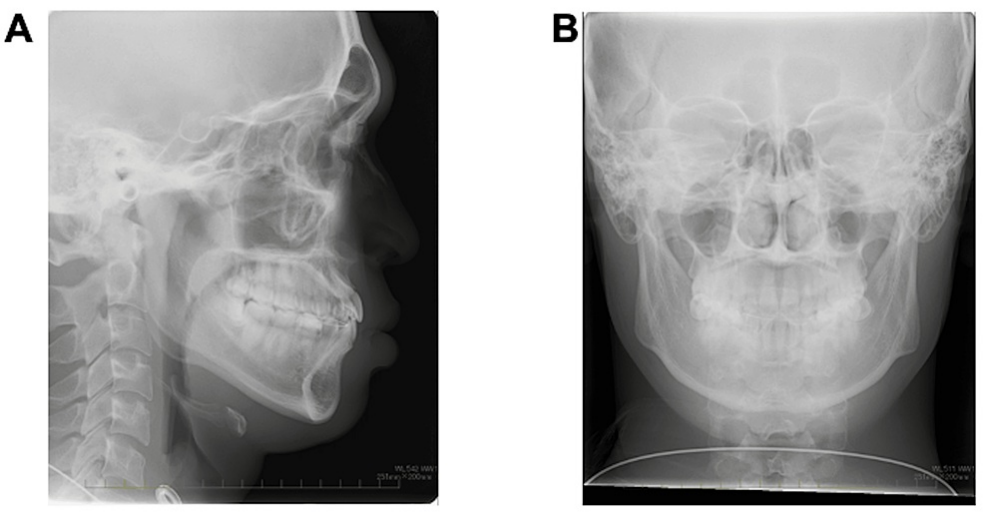
Cephalometric radiographs obtained after orthodontic treatment A: lateral cephalometric radiography performed obtained after orthodontic treatment; B: frontal cephalometric radiography performed after orthodontic treatment

Intraoral photography showed that the overjet improved to 3.0 mm and overbite to 2.0 mm, resulting in an adequate interocclusal relationship between the anterior teeth (Figure 5A). Angle class I occlusal relationship of the molars was maintained. Resin restorations were performed bilaterally for the deciduous maxillary lateral incisors, maxillary canines, and mandibular second molars, and a good occlusal relationship was established (Figure 5A). Panoramic radiography showed no major changes in the periodontal tissues compared to the initial presentation. The bilateral deciduous maxillary lateral incisors, maxillary canines, and mandibular second molars remained well preserved without any root changes (Figure 5B). Resorption of the alveolar bone and recession of the periodontal tissue were observed compared with the start of the retention phase. There was no change in the retroversion of the entire dentition in the oral cavity. The remaining deciduous teeth had not shifted, and no clinical symptoms such as aggravation of periodontitis or spontaneous pain were reported. The following changes were observed in the skeletal system on the lateral cephalometric analysis: the SNB angle increased from 82.6° to 83.2°, the ANB angle changed to -0.3°, and the FMA decreased from 22.4° to 20.0° with a counterclockwise rotation of the mandible (Table 2, Figure 6A). In the dentition, the U1 to SN angle increased from 111.6° to 114.5°, and labial inclination of the maxillary central incisors was noted. The mandibular central incisor showed a lingual inclination with an increase in the FMIA from 64.4° to 69.1°. Although the interincisal angle varied from 125.4° to 126.9°, the angle of inclination of the upper and lower central incisors was within normal limits (Table 2; Figure 6A). Frontal cephalometric analysis showed no significant change relative to the initial visit (Figure 6B). Based on the above-mentioned lateral cephalometric radiographic analyses before and after treatment, the dentition was modified to a condition (dental compensation) in which the maxillary central incisor compensated for the disharmony of the maxillary and mandibular skeleton by the labial inclination and lingual inclination of the mandibular central incisors, and the overjet and overbite were improved. The skeletal change included counterclockwise rotation of the mandible, although the mandible did not grow, and was positioned slightly anterosuperiorly, resulting in horizontal and vertical decreases. The reasons for the counterclockwise rotation of the mandible before and after treatment were as follows: (1) the overjet of the upper and lower incisors increased, and the occlusal contacts and interferences in the upper and lower anterior teeth were removed; and (2) altered occlusal height of the molars due to some mesial migration of the mandibular molars.

**Table 2:** Superimposition of the pretreatment, posttreatment, and six-year post-retention cephalograms SNA: angle formed from the points sella (S), nasion (N), and A; SNB: angle formed from the points S, N, and B; FMA: Frankfort-mandibular plane angle; SN-MP: Sella-Nasion to the mandibular plane angle; IMPA: angle formed by extending the lower incisor long axis to the mandibular plane; FMIA: Frankfort-mandibular incisor angle

Location	Initial	After treatment	After 6 years retention	Norm
SNA	82.7°	82.9°	82.5°	82.8 ± 2.2°
SNB	82.6°	83.2°	83.0°	79.5 ± 2.0°
ANB	0.1°	-0.3°	-0.5°	3.0 ± 1.3°
Facial angle	89.3°	90.5°	90.4°	85.1 ± 3.2°
Y-axis	60.2°	59.7°	59.0°	65.5 ± 3.0°
FMA	22.4°	20.0°	20.5°	25.9 ± 5.9°
SN-MP	29.8°	27.7°	28.7°	32.5 ± 4.9°
Gonial angle	122.3°	121.0°	120.0°	119.6 ± 6.9°
U1 to SN	111.6°	114.5°	115.5°	109.1 ± 6.8°
IMPA	93.2°	90.9°	89.3°	94.0 ± 9.3°
FMIA	64.4°	69.1°	70.2°	60.1 ± 10.2°
Interincisal angle	125.4°	126.9°	126.6°	123.9 ± 8.1°
U1 to A-Pog (mm)	6.8	7.4	7.2	9.6 ± 1.8
L1 to A-Pog (mm)	6.3	4.3	3.9	4.6 ± 2.5
E-line: Upper lip (mm)	2.3	2.4	3.0	2.3 ± 1.3
E-line: Lower lip (mm)	6.0	3.7	5.0	1.7 ± 1.8
Overjet (mm)	0.6	3.0	3.0	3.1 ± 1.1
Overbite (mm)	0.2	2.0	2.0	3.3 ± 1.9

Change After Two Years of Retention

**Figure 7:**
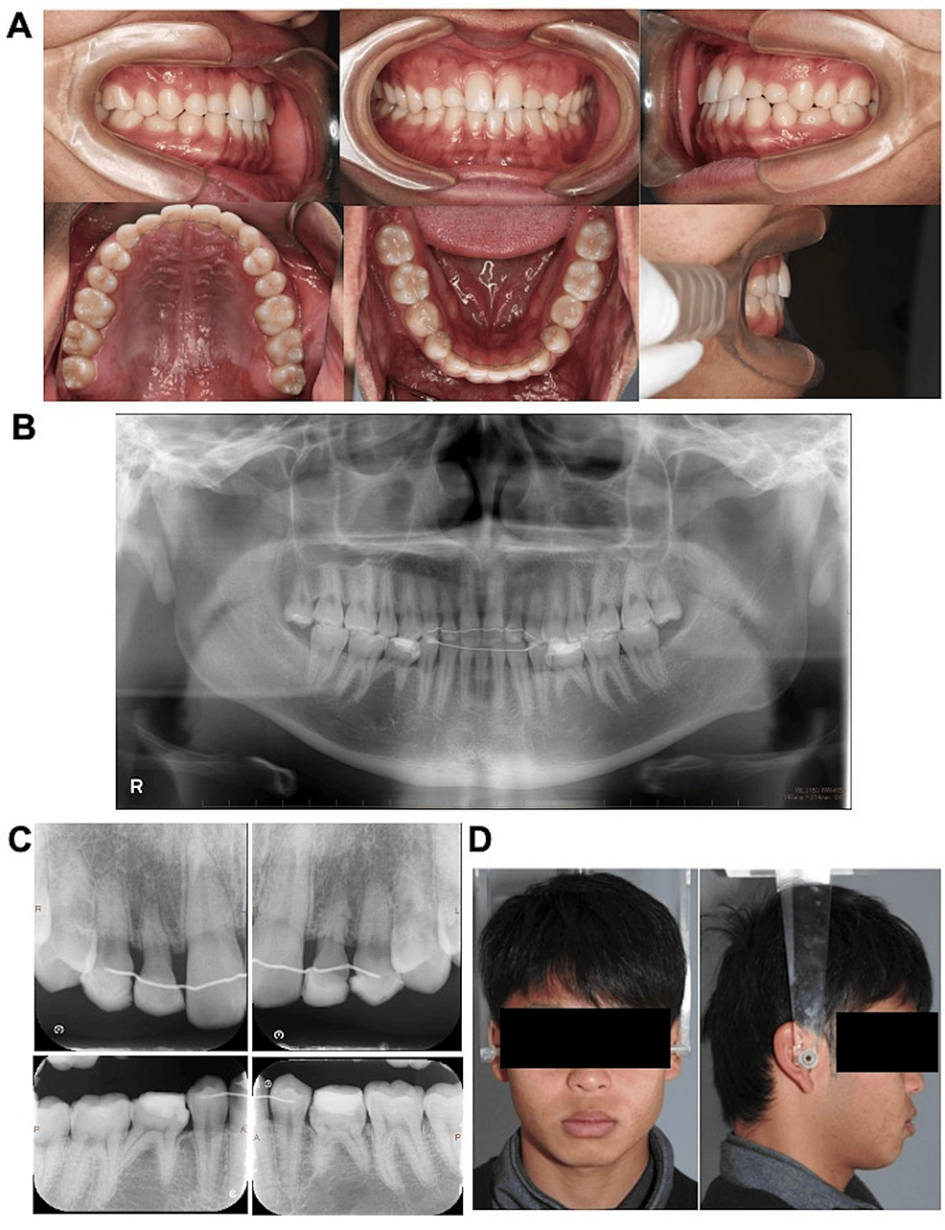
Two years after the start of the retention treatment A: intraoral photographs, B: panoramic radiograph, C: dental radiograph, D: facial photographs

No major changes were observed in the status of the oral cavity at the two-year follow-up compared to that at the end of treatment. Angle class I molar occlusal relationship was maintained, and bilateral resin restorations of the deciduous maxillary lateral incisors, maxillary canines, and mandibular second molars were maintained (Figure 7A). Panoramic and dental radiography showed no major changes in the periodontal tissues relative to those after the end of treatment. Moreover, no changes were observed in the bilateral deciduous maxillary incisors. canines, and the mandibular second molars or in the roots (Figure 7B, 7C). The comparison of the facial photographs at the initial presentation and those acquired at the beginning of retention treatment revealed no notable changes in the facial features (Figure 7D).

Change After Six Years of Retention Treatment

**Figure 8:**
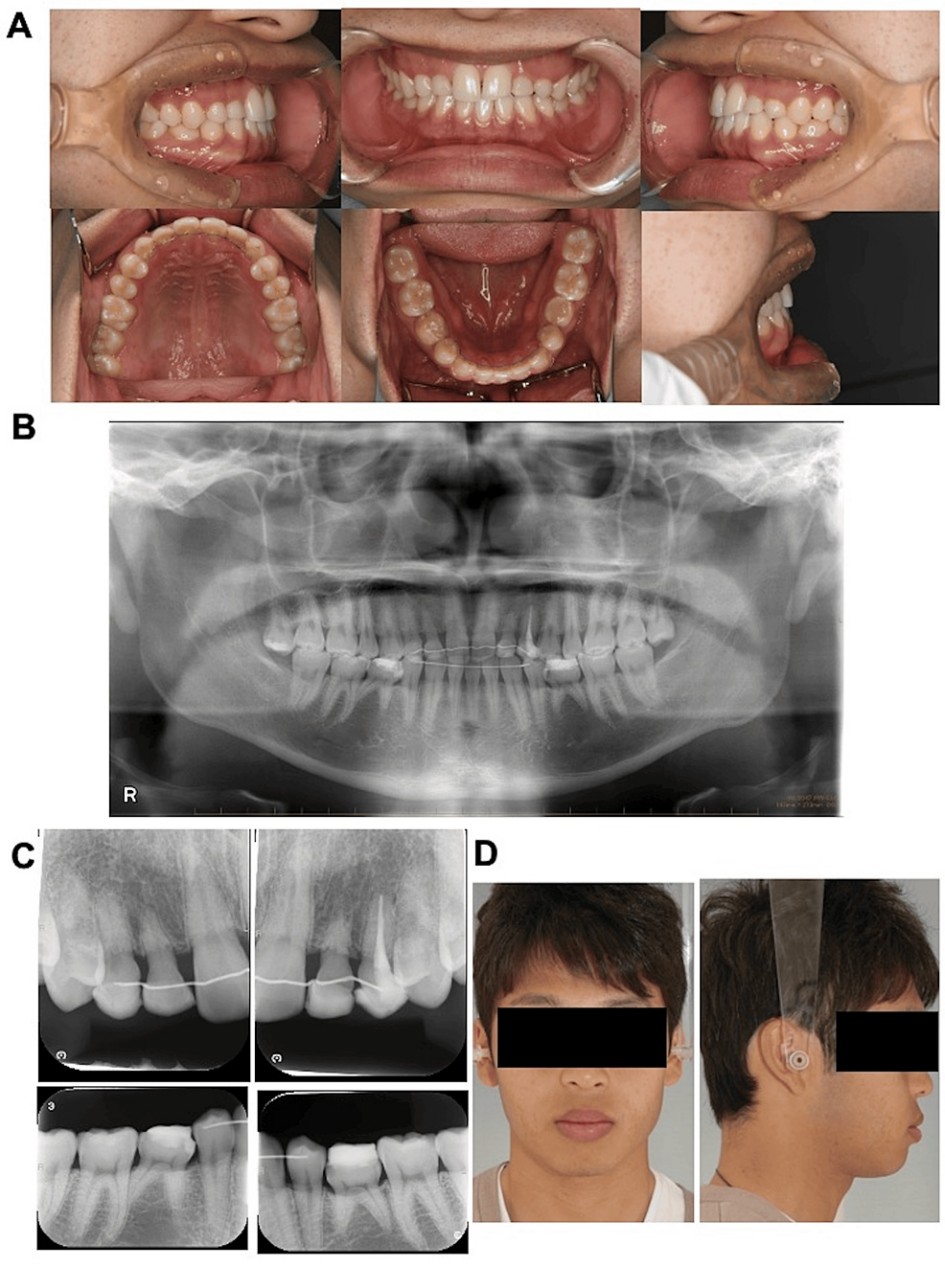
Six years after the retention treatment A: intraoral photographs, B: panoramic radiograph, C: dental radiograph, D: facial photographs

**Figure 9:**
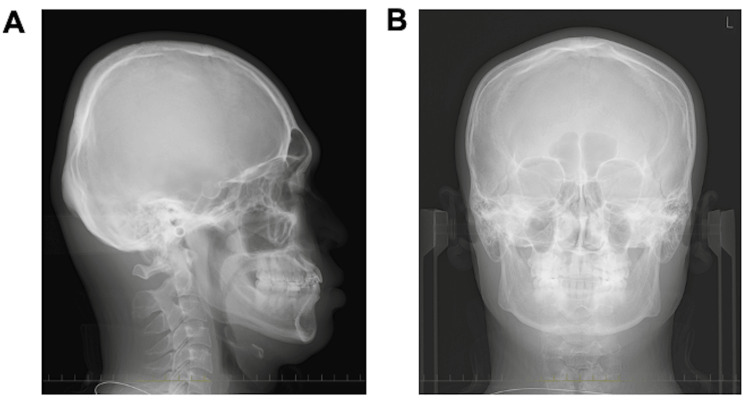
Cephalography performed six years after the retention treatment A: lateral cephalometric radiography obtained six years after the retention treatment, B: frontal cephalometric radiography acquired six years after retention treatment

**Figure 10:**
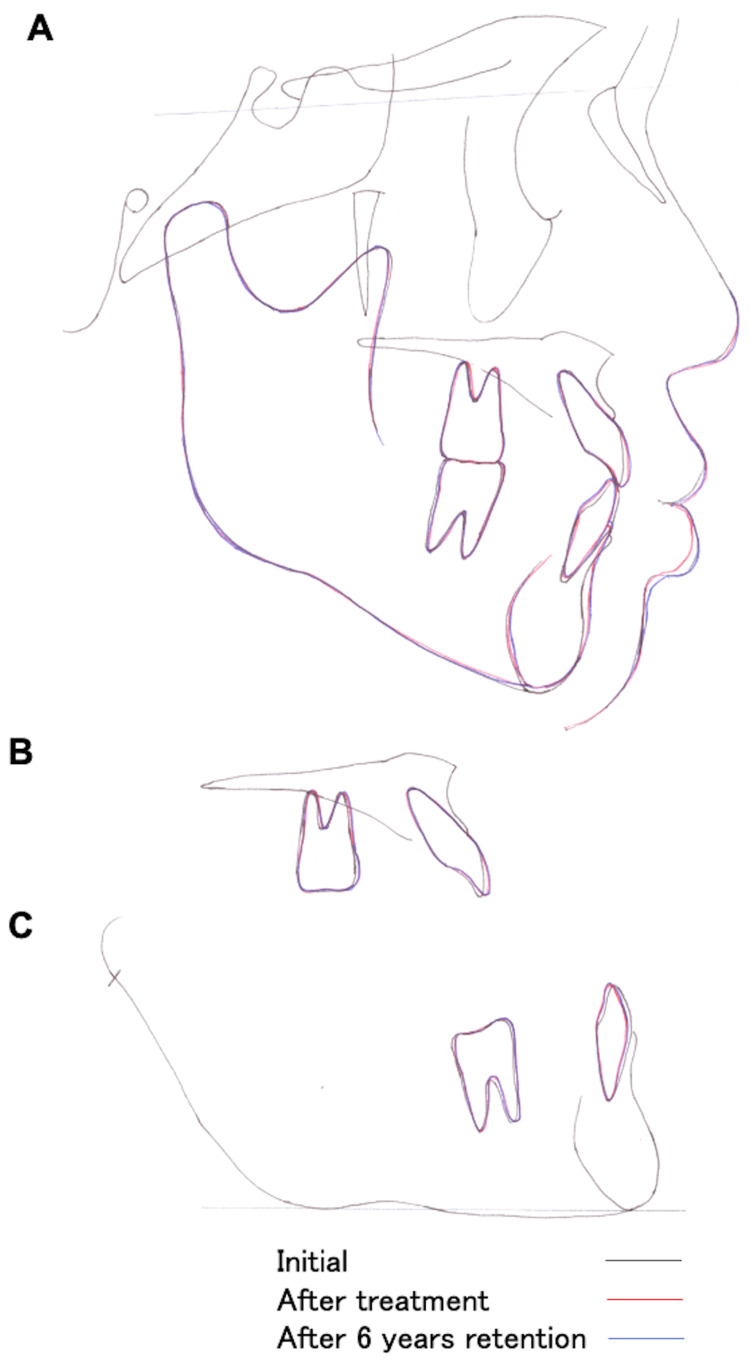
Superimposed tracing of the pretreatment, posttreatment, and six-year post-retention lateral cephalometric radiographs A: overall: SN plane at S; B: maxilla: ANS-PNS at S; C: mandible: mandibular plane at the menton. SN: sella-nasion, ANS: anterior nasal spine, PNS: posterior nasal spine

The intraoral findings at the six-year follow-up did not differ from those observed at the start of the retention phase (Figure 8A). Panoramic and dental radiography showed evidence of an endodontic procedure in the right deciduous maxillary canine, which was absent at the beginning of retention and at the two-year follow-up (Figure 8B, 8C). Root resorption and mild alveolar bone recession were observed with the bilateral deciduous maxillary canines and lateral incisors, and the mandibular second primary molars, which were absent at the initial visit and end of treatment (Figure 8B, 8C). The comparison of the facial photographs obtained at the completion of treatment and two years after the initiation of retention treatment revealed no major changes in both the frontal and profile aspects of the face (Figure 9D). Lateral cephalography did not depict any significant changes in the skeletal system compared to the baseline values (Figures 9A, 10; Table 2). In the dentition, the U1 to SN angle increased from 114.6° to 115.5°, with some changes in the labial inclination of the maxillary central incisors. The FMIA increased from 69.1° at the onset of retention treatment to 70.1° at the six-year follow-up, with a change in the lingual inclination of the mandibular central incisor (Figures 9A, 10; Table 2). Standard frontal radiography did not indicate any change compared to the starting point of retention (Figure 9B).

## Discussion

A systematic review and meta-analysis reported that the prevalence of the congenital absence of one or more permanent teeth was 13.4% in Africa, 7.0% in Europe, 6.3% in Asia, and 5.0% in America [1]. A meta-analysis conducted in Japan suggested that the prevalence of congenital absence of one or more teeth was 5.1% in a general population of approximately 73,000 individuals [6].

The incidence of congenital absence of more than six teeth (oligodontia) is reportedly very low, ranging from 0.08% to 0.16% [7,8], and it varies according to the population type, race, and geographic region. A previous study reported that the prevalence of congenital absence of permanent teeth was the highest for mandibular second premolars, maxillary lateral incisors, and maxillary second premolars [9]. It has been suggested that the differences with respect to the type of missing teeth may depend on race and geographical region. The prevalence of missing mandibular incisors is reportedly higher in East Asia [2,5,10]. An epidemiological investigation on non-syndromic partial edentulism in the Japanese population found that the absence of the maxillary and mandibular second premolars, followed by the maxillary premolars, and the maxillary and mandibular first premolars were relatively more common than the absence of the maxillary lateral incisors, and maxillary and mandibular second molars [11,12]. Thereafter, the absence of only the second premolar and that of both the first and second premolars were the most common patterns in the mandibular dentition [11,12]. By contrast, the prevalence of the pattern involving the absence of the mandibular incisors was unexpectedly low [11,12]. The pattern of missing teeth in the present case included permanent maxillary lateral incisors (bilateral), maxillary canines (bilateral), mandibular left central incisor, and mandibular second premolars (bilateral). Thus, the absence of not only the bilateral mandibular second premolars and permanent bilateral maxillary lateral incisors, which are relatively frequent sites, but also the presence of the bilateral maxillary first and second premolars, as well as the absence of the mandibular right central incisor and bilateral maxillary canines, indicate a rare presentation in this patient, even among cases of non-syndromic partial edentulism.

The effects of non-syndromic partial edentulism on the maxillofacial skeleton include posterior positioning of the maxilla in the horizontal dimension [13] and mandibular prognathism due to the anterior positioning of the mandible [14]. In the vertical dimension, a decreased mandibular inferior border plane angle and reduced anterior cranial height have been reported [13,14]. The effects of non-syndromic partial edentulism on dentition reportedly manifest as a lingual inclination of the mandibular anterior teeth and palatal inclination of the maxillary anterior teeth [2,4,13]. The maxillofacial relationship of this patient was a skeletal class Ⅲ because of the anterior positioning of the mandible, although the relationship of the maxilla to the skull was within the standard range. A decrease in the mandibular inferior border plane angle was also observed. The dentition was characterized by the lingual inclination of the mandibular central incisors, while the inclination of the maxillary central incisors was within the standard values. The palatal inclination of the maxillary central incisors may have been mitigated by the first stage of therapeutic intervention at another hospital. The maxillofacial skeletal morphology of this case showed general agreement with previous studies.

Environmental and genetic factors have been suggested to be responsible for the development of congenital absence of permanent teeth [9]. The environmental factors include trauma, infection, chemotherapy and radiotherapy during infancy, maternal rubella infection during pregnancy, and intrauterine exposure to thalidomide [15]. By contrast, the genetic etiology for non-syndromic partial edentulism includes the involvement of transcription factors, such as MSX1, PAX9 and EDA, EDAR, and TSPEAR in the morphogenesis of WNT10A/B, AXIN2, and the ectoderm in the Wnt-β-catenin pathway [16-25]. Recent years have witnessed the advent of whole-exome analysis techniques with next-generation sequencing. Whole-exosome analysis for Japanese individuals with a family history of non-syndromic partial edentulism suggested that OPN3 is a new candidate causative gene for this condition [26,27]. Identification of susceptibility genes in non-syndromic partial edentulism may enable pre-visit diagnosis, which could facilitate prophylaxis, and early therapeutic intervention could be offered for the predicted malalignment [27]. The search for candidate susceptibility genes for non-syndromic partial edentulism and further elucidation is warranted. 

The patient’s maxillofacial relationship was mandibular prognathism with an ANB angle of 0.1°, making it a borderline case of surgical orthodontic treatment or camouflage treatment. The overjet of the anterior segment was positive and measured 0.6 mm. The axial inclination angle of the maxillary central incisor with respect to the SN and palatal planes, axial inclination of the mandibular central incisor, FMIA value, and axial inclination angle of the maxillary and mandibular central incisors were all within the respective standard values. The maxillomadibular occlusal relationship was angle class I and within the standard range. Cephalometric analysis of the frontal plane did not reveal any major deformity or inclination of the occlusal plane of the mandible. The patient's preferences were thoroughly considered, and non-surgical orthodontic treatment was planned. As described previously, two treatment plans were designed: orthodontic treatment with conservation of the deciduous teeth as much as possible, followed by restorative treatment, and orthodontic treatment with extraction of the deciduous teeth, mesial movement of the maxillary and mandibular molars, and prosthodontic replacement of the missing teeth. A recent case report described the use of a temporary anchorage device in a patient with non-syndromic oligodontia with efficient closure of spaces resulting from the missing permanent teeth [28]. In this case, we also considered extraction of the deciduous teeth, use of orthodontic anchor screws, and mesial movement of the molars. However, the preservation of the remaining deciduous teeth was established as the first choice, and orthodontic treatment was performed, owing to the following factors: (1) the bilaterally over-retained deciduous maxillary lateral incisors and canines, and mandibular second molars were well preserved, (2) absence of tooth mobility or root resorption, (3) the maxillomandibular occlusal relationship was angle class I, and (4) the patient desired treatment with orthodontics and prosthodontic restorations, without surgery. Lateral cephalometric analysis performed at the completion of orthodontic treatment revealed counterclockwise rotation of the mandible and decreased ANB angle compared to the initial visit. Labial inclination of the maxillary central incisors and lingual inclination of the mandibular central incisors improved the position of the maxillary central incisors with respect to the A-Pog (point A-pogonion) plane and that of the mandibular central incisors with respect to the A-Pog plane within normal limits. The overjet improved to 3.0 mm, overbite to 2.0 mm, and an adequate interocclusal relationship was obtained between the anterior teeth, and Angle Class Ⅰmolars occlusal relationship was maintained. Moreover, the extrusion of the right maxillary premolars and bilateral mandibular canines was improved. Thereafter, resin restorations were placed bilaterally on the deciduous maxillary lateral incisors, maxillary canines, and mandibular second molars, resulting in an adequate occlusal stop for the canines and premolars and the establishment of a good occlusal relationship.

In the present case, orthodontic treatment was performed with dental compensation, such that the slope of the anterior teeth of the upper and lower jaws compensated for the skeletal disharmony. The maxillary anterior teeth were shifted labially and the mandibular anterior teeth were shifted lingually, achieving movement that largely aligned with the initial treatment plan. However, the labial inclination of the maxillary and mandibular anterior teeth resulted in lingual inclination. Owing to the patient's pain, we consider that the absence of a full-size angular wire is significant. For torque control, while managing the patient's pain, rectangular wires larger than 0.016 × 0.022 inches should have been installed and torque-controlled efforts should have been made.

At present, six years have passed since the initiation of the retention phase, and notable changes or relapses have not been observed in the maxillofacial skeletal morphology and occlusion. Endodontic treatment was performed for the deciduous right maxillary canine, and root resorption with slight alveolar bone retraction was observed bilaterally in the deciduous maxillary canines, maxillary lateral incisors, and mandibular second molars. However, tooth mobility or symptoms were absent at the last follow-up and the progress was good. However, even in the absence of compression by the existing teeth, over-retained deciduous teeth have been reported to rapidly induce root resorption and may be exfoliated after a certain period of time. Therefore, it is necessary to continue follow-up for the remaining deciduous teeth in the long term and consider the transition to prosthetic treatment with dentures and implants in the event of dislodgement of the deciduous teeth.

The following limitations of this study should be considered. First, the study lacked an adequate sample size, since this study presented a single case report. However, since non-syndromic oligodontia is a rare disease, it may be possible to report only one case at a time. Nevertheless, it is necessary to gather and report more cases in the future and to accumulate more knowledge on this subject. Second, the long-term observation was insufficient, and the longer-term results need to be presented, although this case report provided the results over a six-year retention period. Third, since this is the presentation of one case, potential and unconscious bias may have been present. It is necessary to conduct a prospective study on the effects and long-term comparative examination according to the orthodontic treatment method for patients with non-syndromic oligodontia.

## Conclusions

The treatment of patients with non-syndromic partial edentulism requires highly specialized, comprehensive, and diverse strategies that entail multidisciplinary collaboration between prosthodontics, periodontal conservation, oral surgery, and orthodontics. Non-syndromic partial edentulism is a rare disease, and the variable pattern of occurrence limits the number of cases encountered by a single orthodontist. Therefore, it is important to share information about the treatment plan using case progress reports, including aggregation and long-term progress of the treatment outcomes.
